# Gas-Phase Adsorption of N_2_ on Protonated Molecules and Its Application to the Structural Elucidation of Small Molecules

**DOI:** 10.5702/massspectrometry.A0096

**Published:** 2021-06-10

**Authors:** Hiromori Murashima, Akimasa Fujihara

**Affiliations:** 1 Department of Chemistry, Graduate School of Science, Osaka Prefecture University, Osaka 599–8531, Japan

**Keywords:** electrospray ionization, ion trap, molecular structure, functional group

## Abstract

The gas-phase adsorption of N_2_ on protonated serine (Ser, C_3_H_7_NO_3_), threonine (Thr, C_4_H_9_NO_3_), glycine (Gly, C_2_H_5_NO_2_), and 2-aminoethanol (C_2_H_7_NO) was investigated using a tandem mass spectrometer equipped with an electrospray ionization source and a cold ion trap. N_2_ molecules were adsorbed on the free X–H (X=O and N) groups of protonated molecules. Gas-phase N_2_ adsorption-mass spectrometry detected the presence of free X–H groups in the molecular structures, and was applied to the structural elucidation of small molecules. When the 93 structures with an elemental composition of C_3_H_7_NO_3_ were filtered using the gas-phase N_2_ adsorption-mass spectrometry results for Ser, the number of possible molecular structures was reduced to 8 *via* the quantification of the X–H groups. Restricting and minimizing the number of possible candidates were effective steps in the structural elucidation process. Gas-phase N_2_ adsorption-mass spectrometry combined with mass spectrometry-based techniques has the potential for being useful for elucidating the molecular structures of a variety of molecules.

## INTRODUCTION

Molecular structures are typically identified using nuclear magnetic resonance spectroscopy, X-ray crystallography, optical spectroscopy, and chromatography. Mass spectrometry is routinely used for structural elucidation owing to its high sensitivity, selectivity, and suitability for analyzing mixtures. The elemental compositions of unknown molecules are assigned using high-resolution and accurate mass spectra collected for complex mixtures.^1)^ The number of potential elemental compositions, which increases largely with mass, can be reduced by comparing the experimental and theoretical isotope patterns in mass spectra.^2)^

When the elemental composition of an analyte is elucidated using mass spectrometry, the molecular structure is examined utilizing product ion profiles that are obtained *via* tandem mass spectrometry. The relationships between molecular structure and fragmentation have been studied using a variety of dissociation techniques.^3,4)^ Ion mobility and hydrogen/deuterium exchange have been used to analyze molecular structures.^5,6)^ Nuclear magnetic resonance spectroscopy of gas-phase ions using a magnetic resonance acceleration technique has been developed.^7)^ Mass spectral libraries and electronic databases are typically surveyed for the structural elucidation of unknown molecules in complex mixtures, because the number of potential molecular structures with any elemental composition increases exponentially with mass.^8–10)^

The molecular adsorption of gas-phase ions provides information on the structure of gas-phase ions. N_2_ adsorption on small metal clusters of cations depends on the structure of the cations.^11,12)^ Molecular adsorption experiments on gas-phase carbohydrate ions with 2–5 hydroxy (O–H) groups revealed that the O–H groups of carbohydrates could be quantified using gas-phase N_2_ adsorption-mass spectrometry.^13)^

In this study, we investigated the adsorption of gas-phase N_2_ on protonated molecules using a tandem mass spectrometer equipped with an electrospray ionization source and temperature-controlled ion trap. Gas-phase N_2_ adsorption-mass spectrometry was used for structural elucidation in addition to assigning elemental composition and structural elucidation using fragmentation and mass spectral library surveys with serine (Ser, C_3_H_7_NO_3_), threonine (Thr, C_4_H_9_NO_3_), glycine (Gly, C_2_H_5_NO_2_), and 2-aminoethanol (C_2_H_7_NO) being used as sample molecules.

## EXPERIMENTAL

Gas-phase N_2_ adsorption was detected using a home-built tandem mass spectrometer equipped with an electrospray ionization source and a temperature-controlled 22-pole ion trap (8–350 K).^14,15)^ Protonated molecules were generated *via* the electrospray ionization of solutions containing 0.5 mM of an analyte molecule in a mixture of water and methanol with 1% acetic acid. Analyte molecules were obtained from Nacalai Tesque. The ionization source was operated at a sample flow rate of 3 μL/min, a sheath gas flow rate of 3 L/min, and an applied voltage of 1.5 kV. The protonated molecules were transferred to the gas phase through a metal capillary (340 K) and stored in an octopole ion guide. The gas-phase ions were pulsed into a quadrupole mass filter and a temperature-controlled 22-pole ion trap. The mass-selected ions were thermalized *via* multiple collisions with He containing 30% N_2_ as the buffer gas for 50 ms in the ion trap at 60 K, and the N_2_ molecules were adsorbed on the mass-selected ions in the ion trap. At lower temperatures, the N_2_ molecules condensed on the ion trap electrodes, while at room temperature, the N_2_ molecules were not adsorbed on the ions. The temperature of the ion trap was controlled using a cryogenic refrigerator (CH-204SB, Sumitomo, Japan) and a heater cartridge (HTR-50, Lake Shore, USA), and measured using two silicon diode temperature sensors (DT-670B-CU, Lake Shore, USA). The exact partial pressure and temperature of the N_2_ molecules in the ion trap were not determined in this study, and the ambient pressure in the chamber containing the ion trap was approximately 2×10^−4^ Pa. The maximum numbers of N_2_ molecules that were adsorbed did not depend on the chamber pressure, but the efficiency of N_2_ adsorption was dependent on this. The N_2_-adsorbed cluster ions were extracted from the ion trap and orthogonally accelerated to 2.8 keV using two-stage pulsed electric fields. The ions were mass-analyzed using a reflectron time-of-flight (TOF) spectrometer and counted using dual microchannel plates (F4655, Hamamatsu Photonics, Japan) and a digital storage oscilloscope (104MXi, LeCroy, Japan). The desorption of N_2_ from the weakly bound clusters in the field-free region of the reflectron TOF mass spectrometer was also observed in the TOF mass spectra, in addition to the N_2_-adsorbed cluster ions that were formed in the ion trap.^15)^ The repetition rate of the experimental cycle was 10 Hz.

## RESULTS AND DISCUSSION

### Gas-phase adsorption of N_2_ on protonated molecules

**Figure 1:**
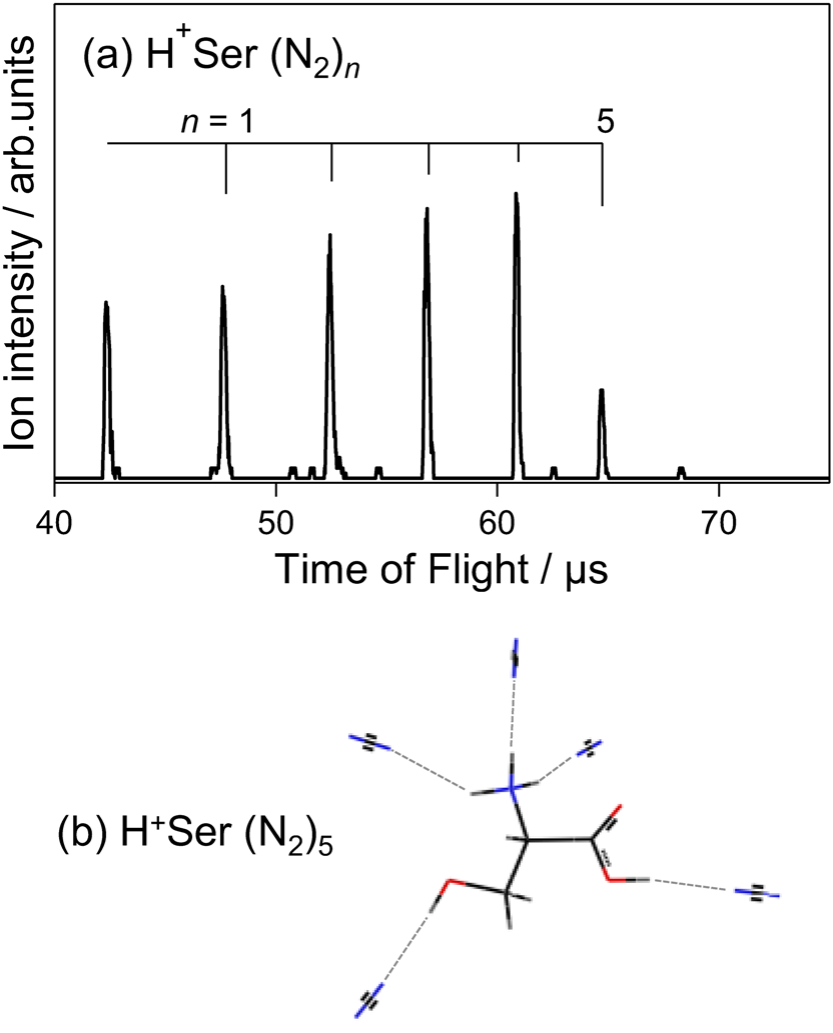
Fig. 1. (a) Mass spectrum of H^+^Ser(N_2_)*_n_*, formed *via* N_2_ adsorption on mass-selected H^+^Ser at 60 K. (b) Optimized structure of H^+^Ser(N_2_)_5_ at the B3LYP/6-31++G(d,p) level, where carbon, nitrogen, oxygen, and hydrogen atoms are colored in black, blue, red, and gray, respectively.

**Figure 2:**
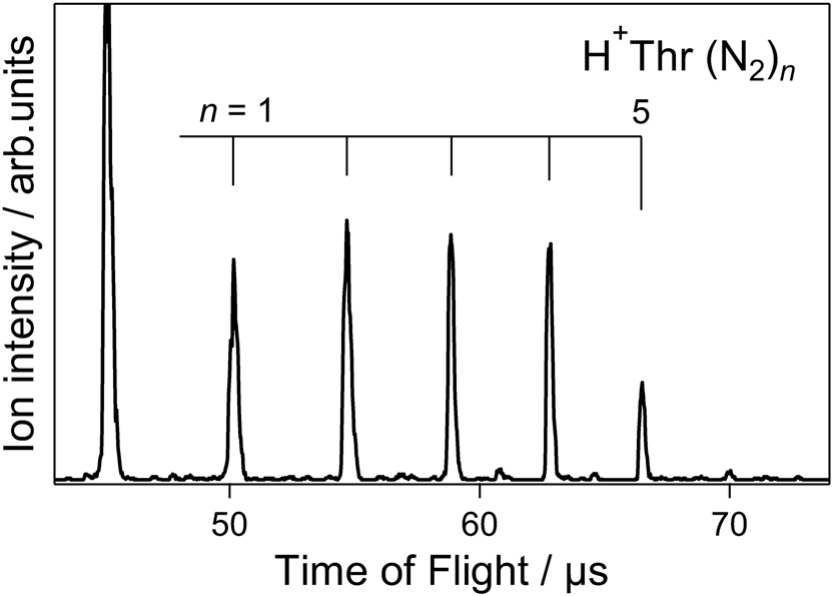
Fig. 2. Mass spectrum of H^+^Thr(N_2_)*_n_*, formed *via* the adsorption of N_2_ on mass-selected H^+^Thr at 60 K.

Figure 1a illustrates the mass spectrum of H^+^Ser(N_2_)*_n_*, which formed *via* the adsorption of N_2_ on mass-selected H^+^Ser at 60 K. Here, *n* denotes the number of N_2_ molecules that are adsorbed on the H^+^Ser in the ion trap, which, according to our experimental results, was *n*≤5. This indicates that the first solvation shell of H^+^Ser consisted of a maximum of five molecules. Stable magic number clusters formed *via* gas-phase N_2_ adsorption on mass-selected ions were observed for carbohydrates and tryptophan (Trp).^13,14)^ It was reported that N_2_ molecules are adsorbed on the H atoms of the O–H groups of carbohydrates, which allowed for the O–H groups of carbohydrates to be quantified using gas-phase N_2_ adsorption data.^13)^ H^+^Trp(N_2_)_5_ was a stable magic number cluster in which five N_2_ molecules were adsorbed on the H atoms of the NH_3_^+^ group, the indole ring, and the carboxyl (COOH) group.^14)^ H^+^Trp(H_2_)*_n_* with *n*=1–5 were reported to be formed in messenger-tagging vibrational predissociation spectroscopic measurements of H^+^Trp using a cryogenic ion trap.^16)^ Diatomic molecules were primarily bound by charge-induced dipole interactions. The optimized structure of H^+^Ser(N_2_)_5_ at the B3LYP/6-31++G(d,p) level using the Gaussian 09 package^17)^ was obtained utilizing previously reported data^14,16,18)^ (Fig. 1b), and the results suggested that five N_2_ molecules were adsorbed on the NH_3_^+^, O–H, and COOH groups of the H^+^Ser. The relative ion intensity of H^+^Ser(N_2_)_5_ was lower than that of H^+^Ser(N_2_)_4_, as illustrated in Fig. 1a. The ion intensity distributions of the N_2_-adsorbed cluster ions indicated that the gas-phase ions had multiple structures.^13)^ The lower relative ion intensity of the *n*=5 cluster was attributed to the intramolecular hydrogen bond between the NH_3_^+^ group and the oxygen atom of COOH group as illustrated in Fig. 1b. In the case of Thr, like Ser, contains an O–H group, the maximum number of adsorbed N_2_ molecules was *n*=5 as illustrated in Fig. 2, indicating that the adsorption sites of H^+^Thr were the NH_3_^+^, O–H, and COOH groups as in the case of H^+^Ser. To reveal the effects of molecular adsorption on the geometric and electronic structures of gas-phase ions, photodissociation spectroscopy and theoretical calculations are required.

**Figure 3:**
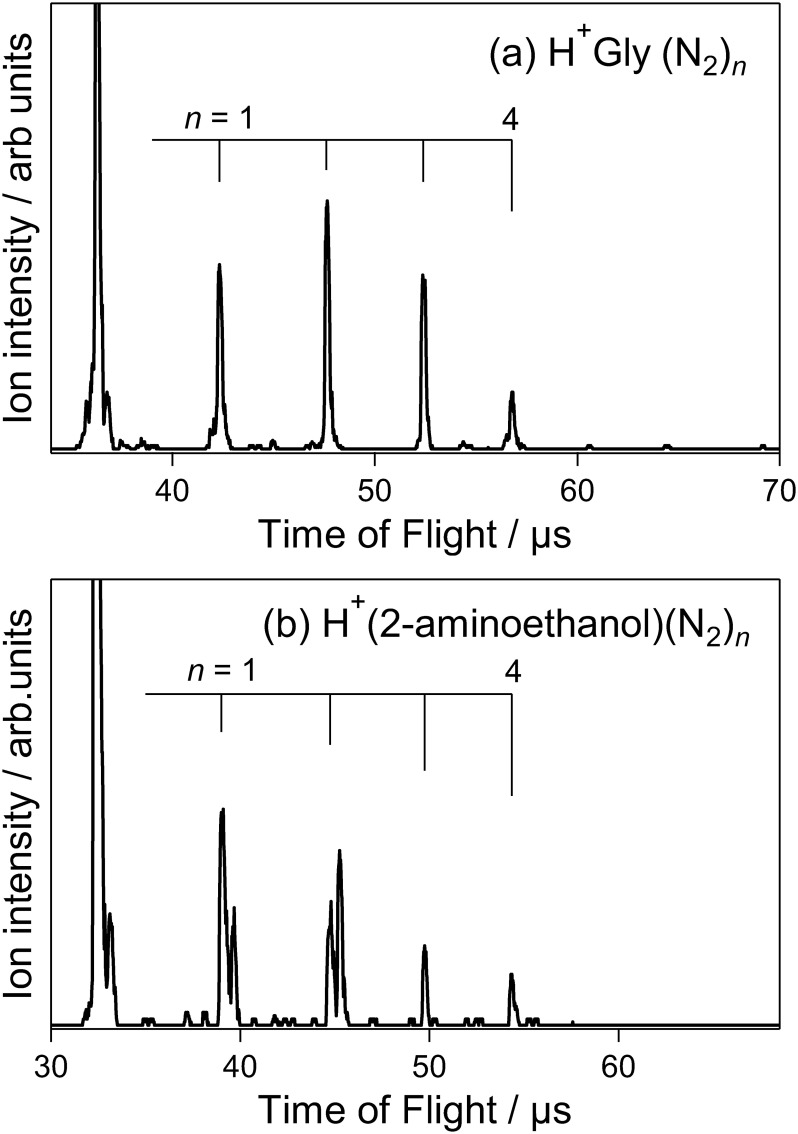
Fig. 3. Mass spectra of (a) H^+^Gly(N_2_)*_n_* and (b) H^+^(2-aminoethanol)(N_2_)*_n_* formed *via* the adsorption of N_2_ on mass-selected H^+^Gly and H^+^(2-aminoethanol) at 60 K, respectively.

To confirm the relationship between N_2_ adsorption and molecular structure, gas-phase N_2_ adsorption-mass spectrometry of H^+^Gly and H^+^(2-aminoethanol) was performed. Gly and 2-aminoethanol lack the O–H and COOH groups of Ser, respectively. Figure 3a illustrates the mass spectrum of H^+^Gly(N_2_)*_n_*, which forms *via* the adsorption of N_2_ on mass-selected H^+^Gly at 60 K. The maximum number of adsorbed N_2_ molecules was *n*=4, and an *n*=5 cluster was not observed in the spectrum. The number of adsorption sites for H^+^Gly was one less than that of H^+^Ser and H^+^Thr. This indicates that four N_2_ molecules were adsorbed on the NH_3_^+^ and COOH groups of H^+^Gly. The maximum number of adsorbed N_2_ molecules for H^+^(2-aminoethanol)(N_2_)*_n_* was also *n*=4 (Fig. 3b), indicating that four N_2_ molecules were adsorbed on the NH_3_^+^ and O–H groups of H^+^(2-aminoethanol). The above findings indicate that gas-phase N_2_ adsorption-mass spectrometry can be used to detect the presence of free X–H (X=O and N) groups in molecular structures.

### Structural elucidation using gas-phase N_2_ adsorption-mass spectrometry

**Figure 4:**
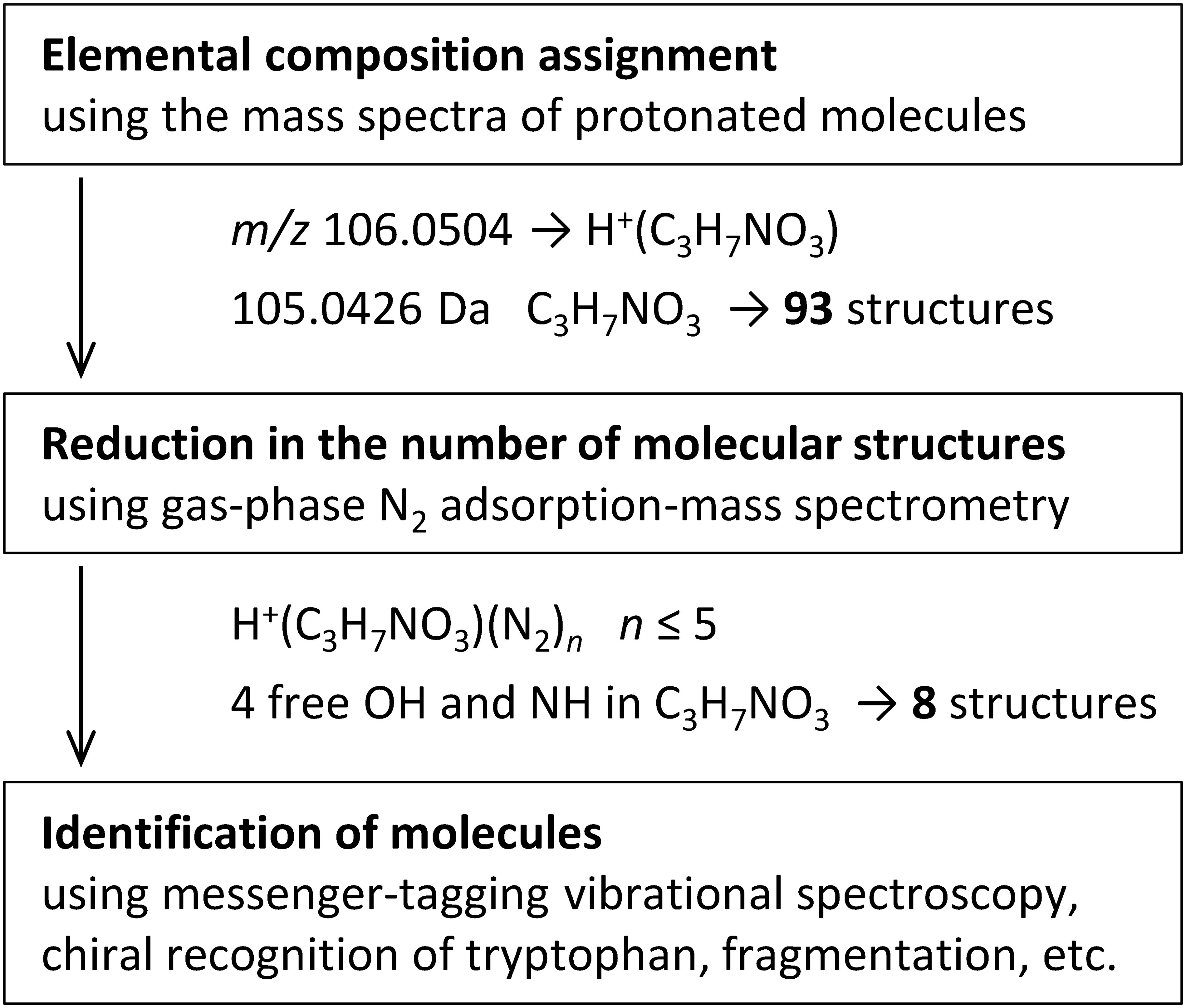
Fig. 4. Strategy for elucidating molecular structures using gas-phase N_2_ adsorption-mass spectrometry.

Based on the aforementioned results, we proposed a method for structural elucidation using gas-phase N_2_ adsorption-mass spectrometry that can be used prior to fragmentation and in surveying mass spectral libraries. Figure 4 illustrates the strategy used for the structural characterization of Ser (C_3_H_7_NO_3_, calculated exact mass 105.0426). The CAS registry revealed 93 possible structures with an emperical formula of C_3_H_7_NO_3_. When conventional methods are used for characterizing molecular structures, the product ion spectrum of the protonated molecule is compared with the product ion spectra in a mass spectral library, and the structures that match the elemental composition and fragmentations are screened.^8-10)^ Restricting and minimizing the number of possible candidates are effective steps of the analysis process.

**Figure 5:**
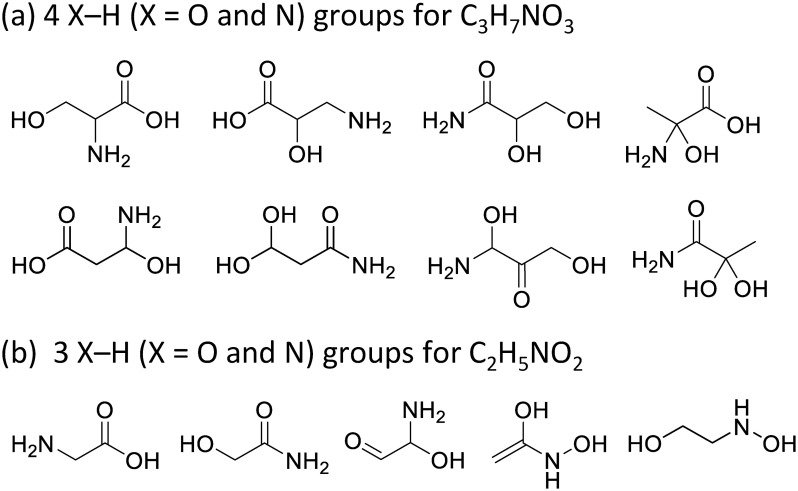
Fig. 5. Potential molecular structures with empirical structures of (a) C_3_H_7_NO_3_ and (b) C_2_H_5_NO_2_.

Gas-phase N_2_ adsorption-mass spectrometry can reduce the number of possible molecular structures with the same elemental composition *via* the quantification of the X–H (X=O and N) groups of the protonated molecule. For the C_3_H_7_NO_3_ elemental composition, the maximum number of N_2_ molecules adsorbed on the protonated molecule was *n*=5, which indicated that C_3_H_7_NO_3_ contained four X–H groups. The number of adsorption sites of C_3_H_7_NO_3_ was derived by subtracting of 1 from the maximum number *n*=5 of H^+^(C_3_H_7_NO_3_), because one N_2_ molecule adsorbed the proton. When the 93 structures associated with the elemental composition of C_3_H_7_NO_3_ were filtered using the number of adsorption sites for C_3_H_7_NO_3_, the number of possible molecular structures was reduced to eight (Fig. 5a). In the case of Gly (C_2_H_5_NO_2_, calculated exact mass 75.0320), the 46 possible structures for C_2_H_5_NO_2_ were reduced to 5 (Fig. 5b) *via* the quantification of the adsorption sites. This method decreased to 8.6 and 10.8% of the number of possible candidates obtained from the conventional methods for C_3_H_7_NO_3_ and C_2_H_5_NO_2_, respectively.

## CONCLUSION

The gas-phase adsorption of N_2_ on protonated molecules generated *via* electrospray ionization was investigated using a tandem mass spectrometer equipped with a temperature-controlled ion trap. This method allowed the number of possible molecular structures with the same elemental composition to be reduced by tenth *via* the quantification of the X–H (X=O and N) groups. Restricting the number of possible candidates is an effective step in the structural elucidation process.

The weakly bound clusters of analyte ions and N_2_ formed in cold ion traps, such as H^+^(C_3_H_7_NO_3_)(N_2_), can be used for the messenger-tagging vibrational spectroscopy analysis of gas-phase analyte ions.^19)^ The optical properties of Trp hydrogen-bonded with analyte ions in the gas phase can be used to identify and quantify the isomers and enantiomers of analyte molecules.^20,21)^ An ion mobility spectrometer with radio-frequency electric fields has been developed for high-resolution and high-transmission efficiency analyses of small molecules.^22)^ The findings reported herein indicate that gas-phase N_2_ adsorption-mass spectrometry combined with mass spectrometry-based techniques can be useful for elucidating molecular structures.

## Notes

Mass Spectrom (Tokyo) 2021; 10(1): A0096

## References

[1] S. Kim, R. P. Rodgers, A. G. Marshall. Truly “exact” mass: Elemental composition can be determined uniquely from molecular mass measurement at ∼0.1 mDa accuracy for molecules up to ∼500 Da. *Int. J. Mass Spectrom.* 251: 260–265, 2006.

[2] N. Stoll, E. Schmidt, K. Thurow. Isotope pattern evaluation for the reduction of elemental compositions assigned to high-resolution mass spectral data from electrospray ionization Fourier transform ion cyclotron resonance mass spectrometry. *J. Am. Soc. Mass Spectrom.* 17: 1692–1699, 2006. 16931035 10.1016/j.jasms.2006.07.022

[3] A. Weissberg, S. Dagan. Interpretation of ESI(+)-MS-MS spectra—Towards the identification of “unknowns.” *Int. J. Mass Spectrom.* 299: 158–168, 2011.

[4] Y. Qi, D. A. Volmer. Structural analysis of small to medium-sized molecules by mass spectrometry after electron-ion fragmentation (ExD) reactions. *Analyst* 141: 794–806, 2016. 26725919 10.1039/c5an02171e

[5] V. Gabelica, A. A. Shvartsburg, C. Afonso, P. Barran, J. L. P. Benesch, C. Bleiholder, M. T. Bowers, A. Bilbao, M. F. Bush, J. L. Campbell, I. D. G. Campuzano, T. Causon, B. H. Clowers, C. S. Creaser, E. De Pauw, J. Far, F. Fernandez-Lima, J. C. Fjeldsted, K. Giles, M. Groessl, C. J. Hogan, S. Hann, H. I. Kim, R. T. Kurulugama, J. C. May, J. A. McLean, K. Pagel, K. Richardson, M. E. Ridgeway, F. Rosu, F. Sobott, K. Thalassinos, S. J. Valentine, T. Wyttenbach. Recommendations for reporting ion mobility mass spectrometry measurements. *Mass Spectrom. Rev.* 38: 291–320, 2019. 30707468 10.1002/mas.21585 PMC6618043

[6] Y. Kostyukevich, T. Acter, A. Zherebker, A. Ahmed, S. Kim, E. Nikolaev. Hydrogen/deuterium exchange in mass spectrometry. *Mass Spectrom. Rev.* 37: 811–853, 2018. 29603316 10.1002/mas.21565

[7] K. Fuke, M. Tona, A. Fujihara, M. Sakurai, H. Ishikawa. Design and development of a novel nuclear magnetic resonance detection for the gas phase ions by magnetic resonance acceleration technique. *Rev. Sci. Instrum.* 83: 085106, 2012. 22938331 10.1063/1.4742768

[8] T. Kind, O. Fiehn. Advances in structure elucidation of small molecules using mass spectrometry. *Bioanal. Rev.* 2: 23–60, 2010. 21289855 10.1007/s12566-010-0015-9 PMC3015162

[9] F. Hufsky, S. Bocker. Mining molecular structure databases: Identification of small molecules based on fragmentation mass spectrometry data. *Mass Spectrom. Rev.* 36: 624–633, 2017. 26763615 10.1002/mas.21489

[10] T. De Vijlder, D. Valkenborg, F. Lemière, E. P. Romijn, K. Laukens, F. Cuyckens. A tutorial in small molecule identification *via* electrospray ionization-mass spectrometry: The practical art of structural elucidation. *Mass Spectrom. Rev.* 37: 607–629, 2018. 29120505 10.1002/mas.21551 PMC6099382

[11] G. N. Patwari, T. Ito, K. Egashira, A. Terasaki. Probing structures of small gold cluster cations with dinitrogen. *Chem. Asian J.* 6: 1834–1838, 2011. 21506280 10.1002/asia.201000901

[12] K. Ohshimo, I. Mizuuchi, K. Akimoto, K. Tsukamoto, M. Tona, H. Yamamoto, M. Nakano, F. Misaizu. Mass spectrometric study of N_2_-adsorption on copper cluster cations formed by modulated pulsed power magnetron sputtering in aggregation cell. *Chem. Phys. Lett.* 682: 60–63, 2017.

[13] N. Ochiai, H. Murashima, A. Fujihara. Quantification of hydroxy groups in carbohydrates using gas-phase N_2_ adsorption. *Chem. Phys. Lett.* 750: 137484, 2020.

[14] A. Fujihara, A. Shimada. Gas-phase N_2_ adsorption on mass-selected hydrogen-bonded cluster ions. *Chem. Phys. Lett.* 718: 1–6, 2019.

[15] A. Fujihara, T. Sato, S. Hayakawa. Enantiomer-selective ultraviolet photolysis of temperature-controlled protonated tryptophan on a chiral crown ether in the gas phase. *Chem. Phys. Lett.* 610–611: 228–233, 2014.

[16] S. Spieler, C. H. Duong, A. Kaiser, F. Duensing, K. Geistlinger, M. Fischer, N. Yang, S. S. Kumar, M. A. Johnson, R. Wester. Vibrational predissociation spectroscopy of cold protonated tryptophan with different messenger tags. *J. Phys. Chem. A* 122: 8037–8046, 2018. 30208709 10.1021/acs.jpca.8b07532

[17] M. J. Frisch, *et al.* Gaussian 09, Revision A.1, Gaussian, Inc., Wallingford, CT, 2009.

[18] R. Wu, T. B. McMahon. An investigation of protonation sites and conformations of protonated amino acids by IRMPD spectroscopy. *ChemPhysChem* 9: 2826–2835, 2008. 18846594 10.1002/cphc.200800543

[19] T. Khuu, N. Yang, M. A. Johnson. Vibrational spectroscopy of the cryogenically cooled *O*- and *N*-protomers of 4-aminobenzoic acid: Tag effects, isotopic labels, and identification of the *E*, *Z* isomer of the *O*-protomer. *Int. J. Mass Spectrom.* 457: 116427, 2020. 32982573 10.1016/j.ijms.2020.116427 PMC7511085

[20] A. Fujihara, N. Maeda. Quantitative chiral analysis of amino acids in solution using enantiomer-selective photodissociation of cold gas-phase tryptophan *via* chiral recognition. *Anal. Chim. Acta* 979: 31–35, 2017. 28599706 10.1016/j.aca.2017.04.027

[21] S. Hanaichi, A. Fujihara. Identification and quantification of leucine and isoleucine residues in peptides using photoexcited tryptophan. *Amino Acids* 52: 1107–1113, 2020. 32710184 10.1007/s00726-020-02875-8

[22] K. Iwamoto, Y. Fujimoto, T. Nakanishi. Development of an ion mobility spectrometer using radio-frequency electric field. *Rev. Sci. Instrum.* 89: 115101, 2018. 30501352 10.1063/1.5050440

